# Food-Derived Elastin Peptides Improve Glucose Metabolism and Protect Renal Vasculature in Stroke-Prone Spontaneously Hypertensive Rats Despite Modest Dipeptidyl Peptidase 4 Inhibition

**DOI:** 10.3390/nu18111759

**Published:** 2026-05-30

**Authors:** Kumiko Takemori, Yuki Nakamura, Kenji Sato, Eri Shiratsuchi, Takashi Kometani, Seiji Masuda

**Affiliations:** 1 Department of Food and Nutrition, Faculty of Agriculture, Kindai University, 3327-204 Nakamachi, Nara 631-8505, Japan; komet03242@gmail.com (T.K.); smasuda@nara.kindai.ac.jp (S.M.); 2 Department of Applied Biological Chemistry, Graduate School of Agricultural Sciences, Kindai University, 3327-204 Nakamachi, Nara 631-8505, Japan; ynakamura7875@gmail.com; 3 Antiaging Center, Kindai University, 3-4-1 Kowakae, Higashiosaka 577-8502, Japan; 4 Agricultural Technology and Innovation Research Institute, Kindai University, 3327-204 Nakamachi, Nara 631-8505, Japan; 5 Division of Applied Biosciences, Graduate School of Agriculture, Kyoto University, Kitashirakawa Oiwake-cho, Sakyo-ku, Kyoto 606-8502, Japan; kenjisato6213@gmail.com; 6 Functional Food Division, Hayashikane Sangyo Co., Ltd., 2-4-8 Yamato-machi, Shimonoseki 750-8608, Japan; esiratsuti@hayashikane.com

**Keywords:** functional food, bioactive peptide, elastin peptide

## Abstract

Background/Objectives: Elastin-derived peptides (EPs) from food sources may be multifunctional dietary components that support metabolic and vascular health. However, their in vivo physiological actions remain incompletely understood. This study investigated the effects of bonito bulbus arteriosus-derived EPs on glucose metabolism, GLP-1 elevation associated with enhanced early-phase insulin secretion, and renal vascular integrity in stroke-prone spontaneously hypertensive rats (SHRSP) with glucose intolerance. Methods: Male SHRSP were administered EPs orally as a single dose (1000 mg/kg) or 4-week regimen (600 mg/kg/day). Glucose tolerance, plasma GLP-1 and insulin levels, and blood glucose levels were measured following glucose loading. Renal morphology was assessed histologically. *Dpp4*, *Icam-1*, and *Agtr1* expression was quantified in glomerular and leukocyte fractions. Leukocyte oxidative signaling was evaluated by quantifying reactive oxygen species production associated with inducible nitric oxide synthase (iNOS). Age-matched Wistar–Kyoto rats were included as normotensive controls. Results: A single dose increased plasma GLP-1 and insulin levels and improved glucose tolerance compared with controls. The 4-week regimen resulted in sustained improvements in glucose tolerance, without changes in blood pressure, a lower nephrosclerosis incidence, and reduced renal and leukocytic inflammatory marker expression. *Dpp4*, *Icam-1*, and *Agtr1* expression was downregulated, and leukocyte iNOS-driven oxidative signaling was reduced. These effects occurred despite the modest DPP4 inhibitory activity of EPs. Conclusions: Food-derived EPs may exert multi-target physiological actions, including GLP-1 elevation with enhanced early-phase insulin secretion and leukocyte oxidative and inflammatory response suppression, which potentially improve metabolic and renal vascular outcomes. EPs warrant further exploratory investigation as candidate functional food ingredients for metabolic and vascular health.

## 1. Introduction

Japan has approximately 350,000 chronic dialysis patients, according to the Japanese Society for Dialysis Therapy Renal Data Registry [1]. A shift in primary renal diagnoses in Japan was reported between 2006 and 2020, during which the number of nephrosclerosis diagnoses increased, whereas that of glomerulonephritis and diabetic nephropathy diagnoses declined [2]. The incidence of nephrosclerosis has surpassed that of glomerulonephritis, making it the second most common cause of chronic dialysis and end-stage renal disease. Hypertensive nephrosclerosis is the leading cause of end-stage renal disease in Japan. Hypertensive renal disease primarily affects the afferent arterioles and glomeruli, and activation of the renin–angiotensin system (RAS) is well established in its pathogenesis [3].

Dipeptidyl peptidase 4 (DPP4; CD26, EC 3.4.14.5) is a homodimeric type II transmembrane glycoprotein with a large extracellular catalytic domain. The soluble form of DPP4 is also present in body fluids, such as plasma and saliva [4]. DPP4 cleaves X-Pro/X-Ala dipeptides from the *N*-terminus of substrates, thereby modulating the activity of various peptide hormones and cytokines [5,6]. DPP4 rapidly inactivates incretins, such as glucagon-like peptide-1 (GLP-1), which regulate postprandial insulin secretion [7]. Several DPP4 inhibitors are widely used to treat type 2 diabetes because they have a low risk of hypoglycemia and neutral effects on body weight [8]. Potential renoprotective effects of DPP4 inhibitors via blood vessel regulation, possibly through increased GLP-1 bioavailability and anti-inflammatory or antioxidant mechanisms, have been reported [9].

Clinically used DPP4 inhibitors are generally well tolerated but can have adverse effects on the gastrointestinal and dermatologic systems [10]. Natural peptides with DPP4 inhibitory activity are being investigated as dietary bioactive compounds with potential metabolic benefits. We previously reported that elastin peptides (EPs) derived from fish bulbus arteriosus exert vasculoprotective effects that improve vascular reactivity without lowering blood pressure in spontaneously hypertensive rats [11]. We also reported that solubilized EPs suppress cellular senescence and inflammation partly via modulation of DPP4 activity [12]. However, the mechanism underlying the blood pressure-independent vascular protective effect of EPs has not yet been fully elucidated.

In this study, we aimed to elucidate the beneficial effects of EPs and the mechanisms underlying their prevention of hypertensive arterial changes without changes in blood pressure.

## 2. Materials and Methods

### 2.1. Materials

Bonito elastin (EPs) was obtained from Hayashikane Sangyo Co., Ltd., Yamaguchi, Japan. The peptide composition of this elastin has been described previously [13]. The EPs contain peptides with molecular weights of less than 1000; their amino acid composition is shown in Table S1.

### 2.2. Animals

Male Wistar–Kyoto (WKY/KPO, WKY) rats were used as normotensive controls, and stroke-prone spontaneously hypertensive rats (SHRSP/KPO, SHRSP) were used as the experimental model (Kindai University, Osaka, Japan) [14]. The animals were maintained at 23 ± 1 °C and 55% ± 5% relative humidity under a 12 h light–dark cycle, housed in standard cages (two rats per cage), and given free access to a Funabashi SP diet (Funabashi Farm Co., Ltd., Chiba, Japan) and water. All animals were acclimatized for 7 days before the experiment. The experimental protocol was approved by the Kindai University Animal Experiment Committee and conducted in strict accordance with the Kindai University Animal Experiment Regulations (approval no. KAAG-2024-003) and ARRIVE guidelines [15].

### 2.3. Peptide Derivative Identification in the Plasma of Rats Administered EPs

EPs (1 g/kg) were administered to SHRSP at 12 weeks of age by oral gavage using a gastric tube. Portal blood samples were collected 5 min after anesthesia induction using syringes treated with 5% EDTA-2Na/0.9% NaCl. Anesthesia was induced using a combination of pentobarbital sodium (Nacalai Tesque Inc., Kyoto, Japan) and isoflurane (FUJIFILM Wako Pure Chemical Co., Osaka, Japan). Blood samples were centrifuged at 16,500× *g* and 4 °C for 10 min to obtain plasma. Plasma was mixed with three volumes of ethanol and centrifuged again at 16,500× *g* and 4 °C for 10 min. The supernatant was used as the deproteinized plasma fraction.

Peptide derivatives detected in rat portal plasma after EP administration were quantified using liquid chromatography–tandem mass spectrometry in multiple reaction monitoring mode using a previously established method [16]. These peptides were selected based on previous reports [13,17] confirming their stability and presence in the systemic circulation after oral elastin ingestion.

### 2.4. Oral Glucose Tolerance Test

The rats were fasted for 15 h before oral administration of a 20% glucose solution (1 mL/100 g body weight) by oral gavage via a gastric tube. The EP-treated group was administered 1 g/kg EPs in a 20% glucose solution. Rats were kept in an incubator (NK-210-3; Natsume Seisakusho Co., Ltd., Tokyo, Japan) at 40 °C for 10 min, and blood samples were collected from the tail vein at specific time (0, 15, 30, 60, and 120 min) intervals following glucose administration. A solution containing 1.5% EDTA-2Na (DOJINDO LABORATORIES, Kumamoto, Japan) in phosphate-buffered saline (PBS; FUJIFILM Wako Pure Chemical Co.) and 100 KIU/mL aprotinin (FUJIFILM Wako Pure Chemical Co.) in 0.9% NaCl was used as an anticoagulant and protease inhibitor. Blood samples were centrifuged at 820× *g* and 4 °C for 10 min, and the resulting plasma was stored at −80 °C until analysis. Plasma GLP-1 levels (GLP-1 ELISA Kit Wako, High Sensitive, 299-75501; FUJIFILM Wako Pure Chemical Co.) and insulin concentrations (LBIS Rat Insulin ELISA Kit, AKRIN-010T; FUJIFILM Wako Shibayagi Co., Gunma, Japan) were measured using commercially available kits. Blood glucose levels were measured using a compact blood glucose meter (Glutest Aqua; Sanwa Kagaku Kenkyusho Co., Ltd., Aichi, Japan).

### 2.5. Evaluation of Hypertensive Renal Failure

#### 2.5.1. Experimental Design

Twelve-week-old male SHRSP (*n* = 19) and WKY (*n* = 9) rats were used. The SHRSP were randomly divided into two groups. One group received a stock chow diet without EPs (Control group; *n* = 9), whereas the other group received a diet with EPs at 600 mg/kg body weight/day (EPs group; *n* = 10), administered twice daily via oral gavage with a gastric tube. The content and weight of administration were chosen because Pro-Gly (PG), the main degradation product of EPs, is metabolized within approximately 4 h [11]. Blood pressure and body weight were measured once weekly. Tail arterial blood pressure was measured using a non-invasive blood pressure monitoring system (BP-98A; Softron Co., Ltd., Tokyo, Japan). The experiment was conducted for 4 weeks. At the end of the experiment, blood samples were collected from the abdominal vena cava under anesthesia using a heparinized syringe (Heparin Sodium Injection-N “AY”; AY PHARMACEUTICALS Co., Ltd., Tokyo, Japan). The brain, heart, and kidneys were excised, weighed, and examined macroscopically. Gross examination of the excised kidneys was performed, and cases showing renal atrophy together with fine granular changes on the renal surface were regarded as nephrosclerosis.

#### 2.5.2. Histological Examination

The right kidney from each rat was fixed in 10% neutral buffered formalin (FUJIFILM Wako Pure Chemical Co.). Paraffin-embedded tissues were sectioned into 2.5 µm-thick slices using a sliding microtome (Sakura Finetek Japan Co., Ltd., Tokyo, Japan), deparaffinized with xylene, and rehydrated through a graded alcohol series. Structural changes in the kidney were evaluated microscopically after hematoxylin–eosin staining. Histological observation was performed using the BZ-H3 application on an All-in-One Fluorescence Microscope (BZ-X800E; Keyence, Osaka, Japan), and the number of vascular necroses per section per individual was counted. To ensure objectivity and methodological rigor, all morphological and pathological assessments were performed in a blinded manner by multiple independent evaluators.

#### 2.5.3. Real-Time Polymerase Chain Reaction

The glomerular fraction from the left kidney was isolated according to the methods described by Yamamoto [18]. The leukocyte-rich fraction (LRF) was isolated from blood as previously described [11]. Total RNA was extracted from both the glomerular and LRFs using the ReliaPrep™ RNA Cell Miniprep System (Promega, Z6010, Madison, WI, USA). Sample optical density was measured at 230, 260, and 280 nm using an e-Spect ES2 Spectrophotometer (BM Equipment Co., Ltd., Tokyo, Japan). Total RNA was reverse-transcribed using the ExScript RT Reagent Kit (Applied Biosystems, Carlsbad, CA, USA) under the following conditions: 25 °C for 10 min, 30 °C for 120 min, 85 °C for 5 s; and stored at 4 °C. Real-time polymerase chain reaction was performed using an ABI PRISM^®^ 7900HT system (Applied Biosystems) with TB Green^®^ Premix Ex Taq™ (Tli RNaseH Plus; Takara Bio Inc., Shiga, Japan) under the following conditions: 95 °C for 30 s (one cycle), followed by 40 cycles of denaturation at 95 °C for 5 s and extension at 60 °C for 30 s. Specific primers were purchased from Takara Bio Inc., and mRNA expression levels were normalized to those of 18S rRNA.

#### 2.5.4. Measurement of Nitric Oxide (NO) Release from the LRF

To determine whether EPs directly modulate inflammatory responses triggered by DPP4, an in vitro stimulation assay was performed using LRF samples isolated from 20-week-old SHRSP (six rats per group). Briefly, LRF samples were pre-incubated with various concentrations of peptides, followed by stimulation with recombinant DPP4 to induce NO production. This setup allowed us to determine the direct immunomodulatory capacity of the peptides against DPP4-induced oxidative signaling.

The fluorescent indicator diaminofluoresceins (DAFs) enable bioimaging of NO production [19]. To measure NO release from leukocytes into the suspension medium, the membrane-impermeable indicator DAF-2 (1 μM; Cayman Chemical Co., Ann Arbor, MI, USA) was added to the LRF suspension (1 × 10^5^ cells/mL in PBS) in a 96-well black plate (SPL Life Science Co., Ltd., Pocheon-si, Republic of Korea) and incubated for 30 min at 37 °C. Fluorescence was measured using a multilabel counter (Wallac ARVO 1420; PerkinElmer Inc., Waltham, MA, USA; excitation 495 nm, emission 515 nm). For inducible NO synthase (iNOS) stimulation experiments, LRFs were treated with 10 ng/mL lipopolysaccharide (LPS), a major component of the outer membranes of Gram-negative bacteria (FUJIFILM Wako Pure Chemical Co.). Furthermore, for DPP4 stimulation or inhibition experiments, LRFs were treated with 2.5 mU/100 µL DPP4 (Sigma-Aldrich Co. LLC., St. Louis, MO, USA). For peptide inhibition experiments using EP hydrolysate products (100 µM), the following peptides were used: H-Gly-Hyp-Gly-OH (GOG; ABclonal Technology, Woburn, MA, USA), H-Val-Pro-OH (VP), cyclo(-Gly-Pro) (cGP; Bachem Holding AG, Bubendorf, Switzerland), cyclo(L-Pro-L-Val) (cPV; Tokyo Chemical Industry Co., Ltd., Tokyo, Japan), and H-Pro-Gly-OH (PG; KOKUSAN CHEMICAL Co., Ltd., Tokyo, Japan). These peptides were added simultaneously to the LRF suspension.

### 2.6. Statistical Analysis

Results are presented as the mean ± standard deviation. IBM SPSS Statistics for Windows, version 29.0 (IBM Corp., Armonk, NY, USA) was used for statistical analysis. For two-group comparisons, Student’s *t*-test or Welch’s *t*-test was applied after assessment of variance equality. For analyses involving three or more groups, one-way analysis of variance (ANOVA) followed by Tukey’s honest significant difference test or Dunnett’s test was used after confirming data normality with the Shapiro–Wilk test. Time-course data, including body weight, blood pressure, blood glucose, GLP-1, and insulin levels, were analyzed using two-way repeated-measures ANOVA to account for time-dependent changes and interactions between treatment and time, followed by a Bonferroni post hoc test. Differences were considered significant at *p* < 0.05.

### 2.7. Artificial Intelligence Declaration

During the preparation of this manuscript, the authors used Microsoft Copilot (Microsoft Corp., Redmond, WA, USA) to proofread the manuscript and enhance its clarity. The authors have reviewed and edited the output and take full responsibility for the content of this publication.

## 3. Results

### 3.1. Quantification of EPs in Portal Vein Plasma Samples After Administration

**Figure 1:**
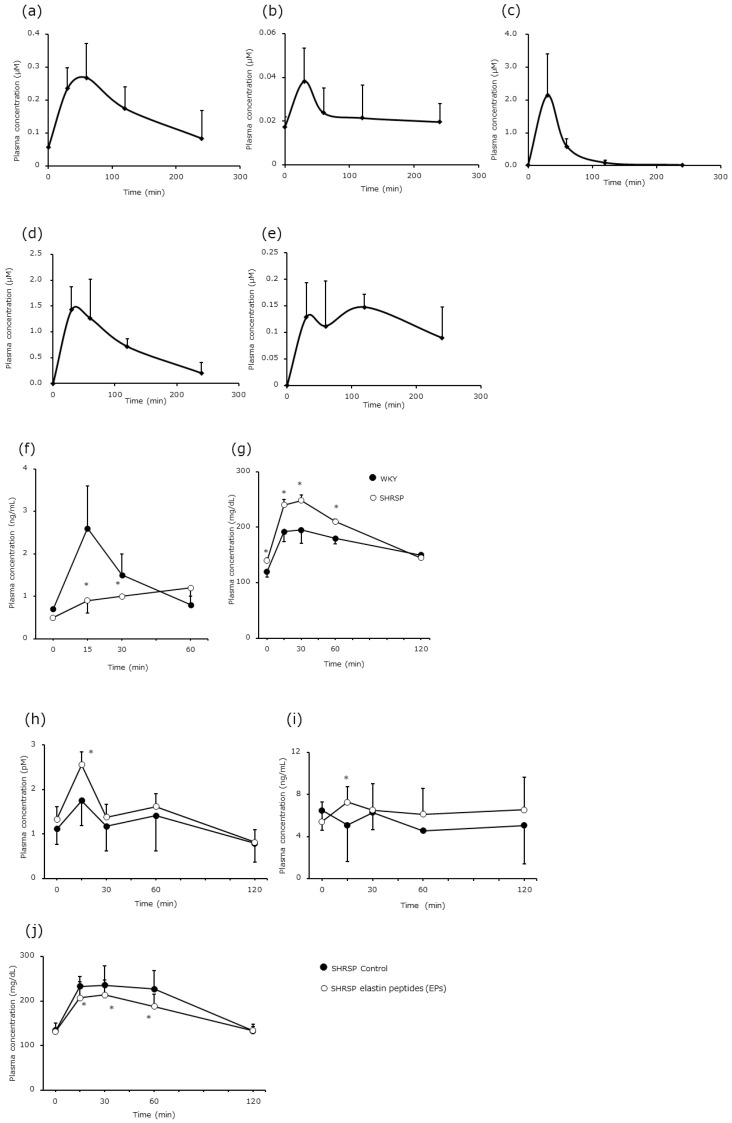
Acute metabolic effects and plasma peptide concentrations following elastin peptide (EP) administration in WKY rats and SHRSP. (**a**–**e**) Plasma concentrations of EP degradation products: (**a**) GOG, (**b**) VP, (**c**) PG, (**d**) cGP, and (**e**) cPV (*n* = 3 per group). (**f**,**g**) Comparison of (**f**) plasma insulin and (**g**) blood glucose levels between WKY rats and SHRSP (*n* = 6 per group). (**h**–**j**) Effects of simultaneous administration of glucose and EPs on (**h**) glucagon-like peptide-1 (GLP-1), (**i**) plasma insulin, and (**j**) blood glucose levels (*n* = 6 per group). Data are presented as the mean ± SD. Statistical analyses for two-group comparisons (**f**–**j**) were performed using Student’s *t*-test or Welch’s *t*-test, as appropriate. * *p* < 0.05 indicate significant differences.

The plasma concentrations of EP degradation products over time following oral administration of EPs in SHRSP were quantified. Five specific peptides—GOG (Figure 1a), VP (Figure 1b), PG (Figure 1c), cGP (Figure 1d), and cPV (Figure 1e)—were detected in the portal vein plasma samples. Their concentrations increased rapidly, peaked between 30 and 60 min, and then gradually decreased.

The DPP4 inhibitory activity of each peptide was evaluated at concentrations corresponding to their plasma levels. The observed inhibitory activities were as follows: GOG (0.25–0.3 µM), 8.73%; VP (0.02–0.04 µM), 11.1%; PG (2 µM), 10.4–11.4%; cGP (1.5 µM), 10.8%; and cPV (0.1 µM), 0.67%. The half maximal inhibitory concentration (IC_50_) values were as follows: sitagliptin, 9.5 × 10^−10^ µM; GOG, 163.2 µM; and VP, 10.68 µM; IC_50_ values could not be determined for PG, cGP, and cPV because of their low inhibitory activity.

### 3.2. Comparison of Strain Differences in Insulin and Glucose Levels

A comparison of plasma insulin and blood glucose levels between WKY rats and SHRSP was performed. In WKY rats, plasma insulin reached its maximum level 15 min after glucose loading, followed by a sharp decrease in blood glucose levels (Figure 1f). In contrast, insulin secretion in SHRSP was significantly lower than that in WKY rats at 15 and 30 min (*p* < 0.05) and slightly increased at 60 min (Figure 1f). Blood glucose levels, from baseline to 60 min after glucose loading, in SHRSP were significantly higher than those in WKY rats (*p* < 0.05); however, the difference disappeared at 120 min (Figure 1g).

### 3.3. Effect of EPs on Glucose Metabolism

The effects of simultaneous administration of glucose and EPs on glucose metabolism were examined. GLP-1 (an incretin) and insulin levels were significantly higher in the EP-treated SHRSP group than in the SHRSP Control group 15 min after glucose loading (*p* < 0.05; Figure 1h,i). Blood glucose levels in the EP-treated SHRSP group increased more slowly than those in the SHRSP Control group and were significantly lower than those in the SHRSP Control group from 15 to 60 min (*p* < 0.05; Figure 1j).

### 3.4. Effects of EP Intake on Hypertensive Renal Failure

#### 3.4.1. Time-Course Changes in Blood Pressure

**Figure 2:**
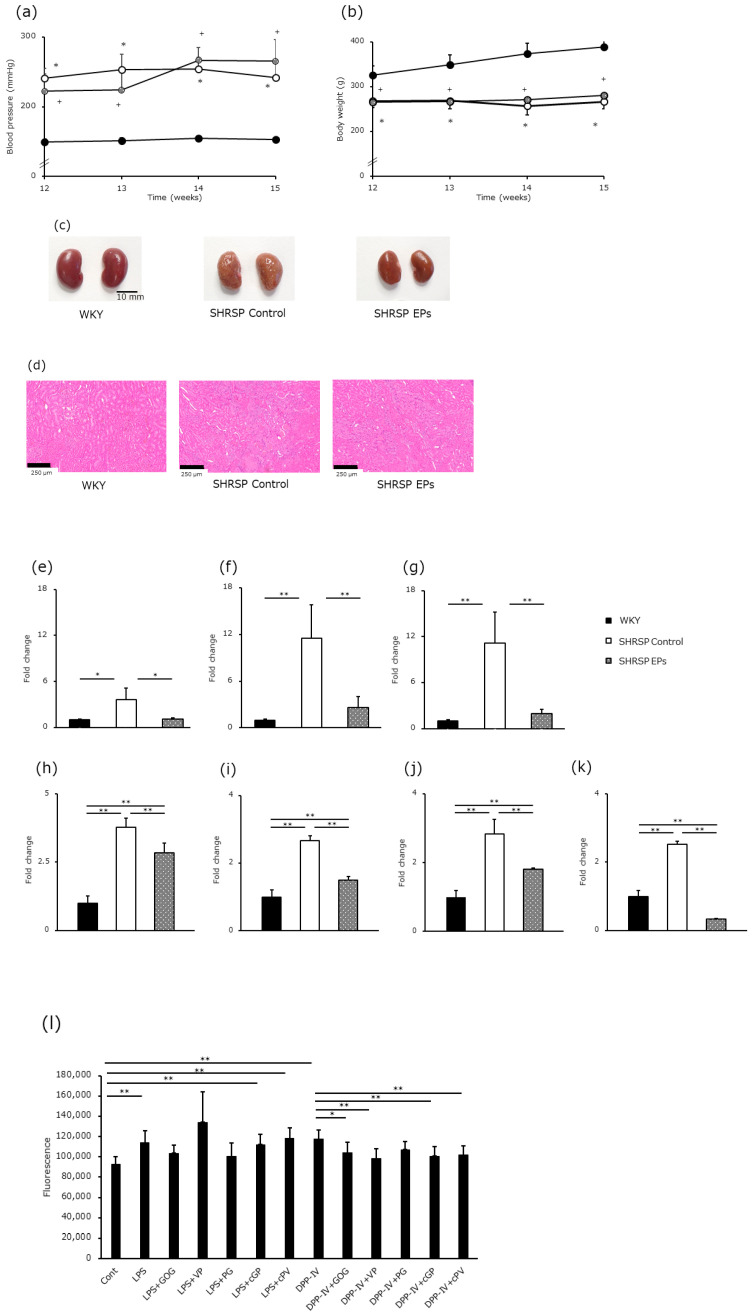
Effects of 4-week EP administration on physiological, morphological, and molecular parameters in SHRSP. (**a**,**b**) Time-course profiles of (**a**) blood pressure and (**b**) body weight. (**c**,**d**) Renal cortical morphology showing (**c**) macroscopic (scale bar: 10 mm) and (**d**) histological (scale bar: 250 µm) features. (**e**–**g**) Glomerular mRNA expression of *Dpp4*, *Icam-1*, and *Agtr1*. (**h**–**k**) Leukocyte-rich fraction (LRF) mRNA expression of *Dpp4*, *Mac-1*, *Agtr1*, and *Nos2*. (**l**) NO production in the LRF. Data represent the mean ± SD (*n* = 9 for WKY, *n* = 9 for SHRSP Control, *n* = 10 for SHRSP EPs for (**a**–**k**); *n* = 6 per group for (**l**)). Time-course data (**a**,**b**) were analyzed by two-way repeated-measures ANOVA. Other comparisons (**c**–**l**) were analyzed using one-way ANOVA followed by Tukey’s or Dunnett’s post hoc test, as appropriate. * *p* < 0.05, ** *p* < 0.01.

Time-course changes in blood pressure and body weight were monitored throughout the experimental period. Blood pressure was significantly higher in both SHRSP groups (Control and EPs) than in the WKY group at all ages (*p* < 0.05; Figure 2a). Body weight throughout the experimental period was significantly lower in both SHRSP groups than in the WKY group (*p* < 0.05; Figure 2b). No significant differences in blood pressure or body weight were observed between the SHRSP Control and EP-treated SHRSP groups, indicating that EP administration did not affect these parameters.

#### 3.4.2. Organ Weight and Morphological Observations

Table 1 shows the organ weights. Although no differences were observed in brain and heart weights among the three groups, kidney weight was significantly lower in the two SHRSP groups than in the WKY group (*p* < 0.01).

No cases of nephrosclerosis were observed in the normotensive WKY rats. In contrast, the SHRSP Control group exhibited distinct cortical atrophy and structural irregularities, which were noticeably attenuated in the EP-treated SHRSP group (Figure 2c,d). Moreover, the number of vascular necroses per section was significantly reduced by EP administration (21 ± 12 vs. 12 ± 12; *p* < 0.05; Figure 2d).

#### 3.4.3. mRNA Expression of *Dpp4*, Intercellular Adhesion Molecule 1 (*Icam-1*), and Angiotensin Receptor Type 1 (*Agtr1*) in the Renal Glomerular Fraction

To estimate the effects of EPs and their degradation products on the expression of factors related to inflammatory and immune responses, we investigated the mRNA expression of the following genes in the renal glomerular fraction: *Dpp4* (Figure 2e), *Icam-1* (Figure 2f), and *Agtr1* (Figure 2g). *Dpp4* mRNA expression in the renal glomerular fraction was significantly higher in the SHRSP Control group than in the WKY group (*p* < 0.05) and significantly lower in the EP-treated SHRSP group than in the SHRSP Control group (*p* < 0.05); *Dpp4* expression was almost suppressed to the same level as that in the WKY group (Figure 2e). The expression patterns of *Icam-1* and *Agtr1* also showed changes similar to those of *Dpp4* (*Icam-1*: WKY vs. SHRSP Control; *p* < 0.01, SHRSP Control vs. SHRSP EPs; *p* < 0.01; *Agtr1*: WKY vs. SHRSP Control; *p* < 0.01, SHRSP Control vs. SHRSP EPs; *p* < 0.01, Figure 2f,g).

#### 3.4.4. mRNA Expression of *Dpp4*, *Mac-1*, *Agtr1*, and *Nos2* in LRF

The expression levels of inflammatory and immune-related factors were further analyzed in circulating leukocytes. The expression levels of *Dpp4*, *Mac-1*, *Agtr1*, and *Nos2* in the LRF were significantly lower in the EP-treated SHRSP group than in the SHRSP Control group (*p* < 0.01; Figure 2h–k).

#### 3.4.5. In Vitro Examination of Leukocyte NO Production

To investigate NO release from leukocytes into the culture medium, fluorescence intensity was measured using a multilabel counter with DAF-2, a membrane-impermeable NO indicator (Figure 2l). To elucidate the mechanisms underlying leukocyte activation, we examined the effects of LPS, DPP4, and EP degradation products on leukocyte stimulation or inhibition. Thirty minutes after DAF-2 addition, the fluorescence intensity in the culture medium of LPS- or DPP4-stimulated leukocytes was significantly higher than that of control leukocytes (*p* < 0.01). Although EP hydrolysate products had no effect on LPS-stimulated NO production, GOG, VP, cGP, and cPV inhibited NO production in DPP4-stimulated leukocytes.

## 4. Discussion

Bioactive peptides derived from dietary proteins have gained increasing attention as multifunctional food components capable of modulating metabolic and vascular functions. Although peptides are often limited by rapid gastrointestinal degradation, accumulating evidence indicates that certain food-derived peptides can reach the circulation and exert physiological effects [17]. In the present study, we demonstrated that EPs from bonito bulbus arteriosus undergo in vivo hydrolysis, enter the bloodstream, and contribute to vascular and metabolic regulation. These findings support the concept that food-derived peptides can act as physiologically relevant modulators rather than simple nutrient sources. The DPP4 inhibitory activity of EPs was modest, indicating that strong enzymatic inhibition alone is unlikely to account for the observed physiological effects. Among dietary proteins, various DPP4 inhibitory peptides have been reported [20]; notably, the VP peptide identified herein exhibited stronger inhibitory activity than previously reported sequences [21]. Despite this relatively limited inhibitory activity, EP administration improved glucose tolerance and increased GLP-1 and insulin levels. However, it must be acknowledged that the present study does not establish a definitive causal link between this modest DPP4 inhibition and the subsequent in vivo metabolic improvements. Because we did not quantify active DPP4 protein in renal tissue or peripheral blood, assess downstream GLP-1 cleavage, or incorporate a GLP-1 receptor antagonist group, the direct pathway of EPs → DPP4 inhibition → higher GLP-1 levels remain unvalidated. Therefore, instead of a singular linear mechanism, we interpret these outcomes as a reflection of the ‘multi-target pleiotropic effects’ inherent to food-derived components. In this context, modest DPP4 inhibition may not act as a primary driver but rather synergizes with other demonstrated pathways, such as the attenuation of inflammatory signaling, reduction in oxidative stress, and modulation of the RAS, to collectively promote metabolic and renoprotective health. Further studies using DPP4-deficient models or specific receptor blockades will be essential to fully substantiate the precise molecular interplay among these integrated pathways.

The insulinotropic and vasculoprotective actions of EPs are better interpreted as pleiotropic effects involving multiple pathways rather than a single dominant mechanism [20]. Additionally, the absence of significant weight reduction suggests that EPs act through pathways distinct from those of GLP-1 receptor agonists, warranting additional investigation into the relative contributions of each mechanism.

We confirmed that circulating EP-derived peptides were associated with suppression of DPP4-mediated leukocyte activation. Sequences containing hydroxyproline, such as GOG, are known to exhibit high resistance to proteolysis and prolonged persistence in vivo [22]. Tripeptides, such as VPP, exert antioxidant, antihypertensive, and gene-regulatory effects [23], whereas cyclic peptides, such as cGP—one of the target peptides in the present study, exhibit high stability and membrane permeability, with potential roles as anti-inflammatory neuropeptides [24]. Herein, the EP-derived peptides selectively suppressed NO production induced by DPP4 stimulation but not by LPS, suggesting pathway-specific rather than broad-spectrum anti-inflammatory activity. This finding aligns with those of Martins et al., who reported that angiotensin II and DPP4 activation contribute to vascular injury through shared inflammatory and oxidative stress pathways [25].

Collectively, these findings highlight that food-derived bioactive peptides function as multi-target modulators of metabolic and vascular homeostasis. EP administration in hypertensive rats enhanced GLP-1 and insulin secretion, improved glucose tolerance, and attenuated renal injury. These effects may be mediated through multiple mechanisms, including incretin regulation, inflammatory marker suppression, and RAS modulation. The pathogenesis of endothelial injury in SHRSP likely involves inflammatory processes, such as leukocyte–endothelial adhesion, which are mediated by salt- [26,27] or angiotensin II-induced hypertension [28,29].

The observed reduction in the expression of *Dpp4*, *Icam-1*, and *Agtr1* in glomeruli, as well as the decreased expression of *Dpp4*, *Mac-1*, and *Agtr1* in leukocytes, substantiated the vasculoprotective and immunomodulatory effects of EPs. This is consistent with prior evidence showing that DPP4 inhibition attenuates renal immune activation, inflammatory adhesion signaling, and RAS-mediated vascular injury [30,31]. The simultaneous downregulation of *Agtr1* and *Icam-1*, alongside *Dpp4*, indicates that EPs exert multifaceted protective effects on the renal microvasculature. These findings suggest that EPs mitigate vascular stress by addressing multiple injurious pathways, including DPP4-mediated signaling, endothelial inflammation, and RAS overactivation.

Our in vitro findings that EP-derived peptides suppress NO production in DPP4-stimulated LRF suggest that these peptides possess direct anti-inflammatory properties. This supports the idea that the vascular protection observed in vivo may be partly due to the direct suppression of DPP4-mediated inflammatory signaling in circulating immune cells. Furthermore, the pharmacological distinction between food-derived components and established synthetic DPP4 inhibitors, such as sitagliptin, is worth noting. While synthetic pharmaceuticals are designed for high target specificity, distinct bioavailability, and sustained-release profiles to strongly inhibit a single enzyme, food-derived bioactive peptides, such as the EPs evaluated here, typically exert mild, complex, and pleiotropic effects across multiple physiological pathways. Our primary objective was to evaluate the integrated, multi-target efficacy of EPs as a functional food ingredient rather than a single-target pharmaceutical. This holistic approach, supported by recent literature on cardiorenal protection by dietary peptides [32,33,34,35], provides a realistic representation of how food-derived components contribute to overall metabolic and vascular health.

This study has some limitations. First, only one disease model (SHRSP) was used, and the treatment period was limited to 4 weeks at a relatively high dose (600 mg/kg). As SHRSP inherently exhibit glucose intolerance, it is difficult to fully exclude the contribution of improved glucose metabolism to the observed renoprotective effects. Using hypertensive models without metabolic abnormalities would provide clearer mechanistic insights. Second, the evaluation of renal protection was primarily based on histological and molecular markers at an early stage of hypertension-induced injury. Due to the 4-week experimental duration, which may be insufficient for manifesting overt renal failure, functional renal markers, such as serum creatinine, eGFR, or proteinuria, were not determined. While these molecular and histological markers provide strong evidence of protection, comprehensive functional evaluation of vascular health, such as assessment of pulse wave velocity or direct vascular reactivity, was not performed. Therefore, simply reporting stable systolic/diastolic blood pressure is insufficient to fully substantiate a pressure-independent protective mechanism. Longer-term studies evaluating quantitative renal function markers, dose-response relationships at lower intakes, and vascular function are required to validate the clinical relevance of EPs in humans. Additionally, our pharmacokinetic peptide quantification was performed in a small sample group (*n* = 3) without reporting effect sizes or confidence intervals, which limits the statistical generalization of the absorption kinetics; these preliminary parameters should be validated in larger-scale profiles.

## 5. Conclusions

Although EPs from bonito bulbus arteriosus exhibited only modest DPP4 inhibitory activity, they improved glucose metabolism and attenuated renal vascular injury in SHRSP, suggesting potential pleiotropic metabolic and vascular effects. These results underscore the potential of food-derived peptides as bioactive compounds that may contribute to metabolic and vascular regulation. Rather than acting through strong DPP4 inhibition or GLP-1 receptor agonism, EPs appear to modulate multiple interconnected physiological pathways, including incretin signaling, inflammatory responses, and RAS-related mechanisms. Accordingly, EPs may hold promise as multi-target functional food ingredients for supporting metabolic and renal health, rather than as therapeutic agents. Moreover, multiple EP-derived peptides were detected in the bloodstream, but their stability and bioavailability require optimization to achieve consistent physiological effects.

Notably, these preclinical findings are obtained in an animal model. To transition beyond the current preclinical stage and establish clinical readiness, concrete next steps are required. Future investigations will focus on: (a) preliminary human pharmacokinetic and bioavailability assessments to determine exposure levels; (b) in vitro feasibility studies utilizing the human gut microbiota to understand peptide degradation and metabolism; and (c) initial phase I safety trials in humans. These integrated translational steps are essential to validate the practical intake levels and therapeutic efficacy of EPs for human cardiorenal health.

## Figures and Tables

**Table 1:** Comparison of organ weights.

Organ	WKY	SHRSP Control	SHRSP EPs
Brain	1.923 ± 0.041	2.048 ± 0.112	1.981 ± 0.132
Heart	1.276 ± 0.042	1.351 ± 0.071	1.233 ± 0.218
Kidney (right)	1.410 ± 0.089	1.091 ± 0.117 **	1.013 ± 0.155 **
Kidney (left)	1.351 ± 0.122	1.118 ± 0.079 **	1.029 ± 0.171 **

Mean ± standard deviation (SD), Dunnett’s test; ** *p* < 0.01 vs. WKY. Elastin peptides (EPs); stroke-prone spontaneously hypertensive rats (SHRSP); Wistar–Kyoto rats (WKY/KPO). Values are shown in grams.

## Data Availability

The data presented in this study are available on request from the corresponding author.

## Supplementary Materials

The following supporting information can be downloaded at: https://www.mdpi.com/article/10.3390/nu18111759/s1, Table S1: Amino acid composition of bonito elastin peptide (residues/1000 residues).

## Abbreviations

The following abbreviations are used in this manuscript:

*Agtr1*	angiotensin receptor type 1
cGP	cyclo(-Gly-Pro)
cPV	cyclo(L-Pro-L-Val)
DAF	diaminofluorescein
DPP4	dipeptidyl peptidase 4
EPs	elastin peptides
GLP-1	glucagon-like peptide-1
GOG	H-Gly-Hyp-Gly-OH
IC_50_	half maximal inhibitory concentration
*Icam-1*	intercellular adhesion molecule 1
iNOS	inducible NO synthase
LPS	lipopolysaccharide
LRF	leukocyte-rich fraction
*Mac-1*	CD11b/CR3
NO	nitric oxide
*Nos2*	nitric oxide synthase 2
PBS	phosphate-buffered saline
PG	H-Pro-Gly-OH
RAS	renin–angiotensin system
SHRSP	stroke-prone spontaneously hypertensive rats
VP	H-Val-Pro-OH
WKY/KPO	Wistar–Kyoto rats
