# Supplementary material for: Food-Derived Elastin Peptides Improve Glucose Metabolism and Protect Renal Vasculature in Stroke-Prone Spontaneously Hypertensive Rats Despite Modest Dipeptidyl Peptidase 4 Inhibition
Source: Nutrients. 2026 May 30;18(11):1759. doi: 10.3390/nu18111759 (PMC13258851; doi:10.3390/nu18111759)
Supplement: Supplementary file 1 [file nutrients-18-01759-s001.zip › nutrients-4311275-supplementary.pdf]

Supplementary Material

Supplementary Table S1. Amino acid composition of bonito elastin peptide (residues/1000 residues)

Amino acid	Elastin peptide from skipj	Literature value (elastin from yellowtail fish)
Asp	21	18
Thr	68	63
Ser	25	32
Glu	37	33
Gly	440	400
Ala	85	130
Val	72	57
Cys	1	<0.6
Met	4	6.4
Ile	12	12
Leu	37	37
Tyr	30	38
Phe	21	31
Hyl	0	0
His	3	3.5
Lys	8	11
Ide	0.3	0.4
Des	0.2	0.4
Arg	25	21
Hyp	7	8.5
Pro	102	99

Asp, aspartic acid; Thr, threonine; Ser, serine; Glu, glutamic acid; Gly, glycine; Ala, alanine; Val, valine; Cys, cysteine; Met, methionine; Ile, isoleucine; Leu, leucine; Tyr, tyrosine; Phe, phenylalanine; Hyl, hydroxylysine; His, histidine; Lys, lysine; Ide, insulin-degrading enzyme; Des, desmosine; Arg, arginine; Hyp, hydroxyproline; Pro, proline.
